# A novel noninvasive surface ECG analysis using interlead QRS dispersion in arrhythmogenic right ventricular cardiomyopathy

**DOI:** 10.1371/journal.pone.0182364

**Published:** 2017-08-03

**Authors:** Wan-Hsin Hsieh, Chin-Yu Lin, Abigail Louise D. Te, Men-Tzung Lo, Cheng-I Wu, Fa-Po Chung, Yi-Chung Chang, Shih-Lin Chang, Chen Lin, Li-Wei Lo, Yu-Feng Hu, Jo-Nan Liao, Yun-Yu Chen, Shih-Jie Jhuo, Sunu Budhi Raharjo, Yenn-Jiang Lin, Shih-Ann Chen

**Affiliations:** 1 Division of Cardiology, Department of Medicine, Taipei Veterans General Hospital, Taipei, Taiwan; 2 Institute of Translational and Interdisciplinary Medicine and Department of Biomedical Sciences and Engineering, National Central University, Chung-Li, Taiwan; 3 Faculty of Medicine and Institute of Clinical Medicine, National Yang-Ming University, Taipei, Taiwan; 4 Department of Medicine, Taipei Veterans General Hospital, Yuan-Shan Branch, I-Lan, Taiwan; 5 HB Calleja Heart and Vascular Institute, St. Luke’s Medical Center, Quezon City, Philippines; University of Minnesota, UNITED STATES

## Abstract

**Background:**

This study investigated the feasibility of using the precordial surface ECG lead interlead QRS dispersion (IQRSD) in the identification of abnormal ventricular substrate in arrhythmogenic right ventricular cardiomyopathy (ARVC).

**Methods:**

Seventy-one consecutive patients were enrolled and reclassified into 4 groups: definite ARVC with epicardial ablation (Group 1), ARVC with ventricular tachycardia (VT, Group 2), idiopathic right ventricular outflow tract VT without ARVC (Group 3), and controls without VT (Group 4). IQRSD was quantified by the angular difference between the reconstruction vectors obtained from the QRS-loop decomposition, based on a principal component analysis (PCA). Electroanatomic mapping and simulated ECGs were used to investigate the relationship between QRS dispersion and abnormal substrate.

**Results:**

The percentage of the QRS loop area in the Group 1–2 was smaller than the controls (P = 0.01). The IQRSD between V1-V2 could differentiate all VTs from control (P<0.01). Group 1–2 had a greater IQRSD than the Group 3–4 (V4-V5,P = 0.001), and Group 1 had a greater IQRSD than Group 3–4 (V6-Lead I, P<0.001). Both real and simulated data had a positive correlation between the maximal IQRSD (γ = 0.62) and the extent of corresponding abnormal substrate (γ = 0.71, both P<0.001).

**Conclusions:**

The IQRSD of the surface ECG precordial leads successfully differentiated ARVC from controls, and could be used as a noninvasive marker to identify the abnormal substrate and the status of ARVC patients who can benefit from epicardial ablation.

## Introduction

Ventricular arrhythmias (VAs) arising from the right ventricular outflow tract (RVOT) are usually considered “idiopathic” if there are no significant structural abnormalities, and are “benign cardiac diseases”; radiofrequency ablation can be effective for symptomatic control if medical treatment is refractory [1]. Arrhythmogenic right ventricular cardiomyopathy (ARVC) is an inherited cardiomyopathy characterized by pathological fibrofatty infiltration and cardiomyocyte (CM) loss predominantly in the right ventricle (RV), leading to heart failure and lethal arrhythmias [2–6]. The recent cellular study demonstrated a metabolic derangement may relate to ARVC pathologies, indicating a different underlying mechanism of the disease [7,8]. The manifestation of ARVC patients was mostly associated with ventricular arrhythmias (VAs) from the RV that can eventually lead to heart transplantation and cardiac death. Also, sustained atrial arrhythmias had been recognized as common clinical arrhythmias [9–11]. The structural, pathological, and electrophysiological abnormalities of early ARVC may be subtle and difficult to distinguish from idiopathic VAs from the RVOT [12,13]. It is well known that the change of T wave in precordial leads V1 to V3 during normal sinus rhythm (SR) facilitates the diagnosis of ARVC and indicates potential abnormal substrates beneath the precordial leads, but recent data have shown that these criteria may not be reliable due to low sensitivity [14–16]. Peters et al. demonstrated that an increased dispersion of the QRS complex, especially in the precordial leads, was a predictor of recurrence of VAs and a risk factor for sudden cardiac death [17,18]. Since the epicardial reentry circuit was associated with ARVC arrhythmia, the noninvasive electrocardiogram (ECG) is important to identify diseased substrates of ARVC.

We hypothesized that the spatiotemporal ECG depolarization dispersion of QRS would be a sensitive noninvasive marker for detecting the substrate abnormality, especially when a potential abnormal substrate emerges beneath the precordial leads. The characteristics of surface ECG during SR were systematically analyzed and their association with substrate abnormality was explored. In this study, we investigated the surface ECG in consecutive definite ARVC patients with or without dominant epicardial abnormal substrate and compared the results with those obtained from patients without abnormal substrate of the RV. Data was confirmed by invasive electrophysiological study. For the comparison, patients of without inducible ventricular tachycardia (VT) were included as the control group. Particularly, we investigated the depolarization abnormalities in terms of interlead QRS dispersion (IQRSD) from surface ECG among the precordial leads, where the data were derived from a reconstructed ECG that was decomposed by principal component analysis (PCA). We also used a simulation model to examine and analyze the variation in the surface ECG with respect to the scar on the right ventricular epicardium.

## Material and methods

### Study population

This study complies with the Declaration of Helsinski, and the study protocol was approved by the Institutional Review Board (IRB) in Taipei Veterans General Hospital, (IRB number: 2013-07-034B) without the requirement of the written or verbal informed consent. Between March 2000 and January 2013, a total of 71 consecutive patients were enrolled, including 51 consecutive patients with drug-refractory symptomatic VAs from the RV detected by surface ECG (Group 1–3) and 20 control patients (Group 4, control group). These symptoms and findings included left bundle branch block-like morphology and inferior axis, frequent (>20%/day) ventricular premature contractions, and occurrence of sustained or non-sustained VT. All the enrolled subjects were divided into four groups: Group 1: definite ARVC fulfilling the Task Force (TF) criteria and showing epicardial abnormal substrate (> 20% of the low voltage zone (LVZ, peak-to-peak bipolar voltage of 0.5 mV) of the epicardium) and the requirement of epicardial ablation; Group 2: definite ARVC fulfilling the TF criteria with abnormal substrate who did not require epicardial ablation; Group 3: idiopathic RVOT-VT not fulfilling the TF criteria; and Group 4: atrial ventricular nodal reentrant tachycardia (AVNRT, n = 20) without any inducible VT, as an age-sex matched control during the concurrent period. The definitions of an abnormal RV structure/image, depolarization abnormalities of Epsilon wave in the surface ECG or late potentials by signal-averaged ECG (SAECG), repolarization abnormalities of T wave inversion (TWI) in the surface ECG, pathological fibrofatty infiltration in tissue characterization of RV wall, arrhythmias from the RV outflow configuration with left bundle-branch block morphology (LBBB), and family history of ARVC or unexplained sudden cardiac death in first degree relatives or identification of genetic mutation, were based on the revised TF consensus and were well documented in the major and minor criteria (Table 1) [19]. The filtered QRS duration of >114 ms, the duration of high-frequency and low-amplitude signal in the terminal portion of the filtered QRS complex of ≧38 ms, and the root mean square voltage of the terminal portion of the filtered QRS complex of <20 uV were the SAECG criteria for ARVC [20]. Patients with aberrant conduction at baseline were not included in this study.

**Table 1:** Revised task force criteria in different groups of patients.

Characteristics	Definite ARVC with epicardial ablation, Group 1 (N = 10)	Definite ARVC without epicardial ablation, Group 2 (N = 16)	Idiopathic RVOT VT, Group 3 (N = 25)	P value
**Depolarization**				
Minor	7(70%)	9(56.3%)	2(8%)	<0.001
Major	3(30%)	3(18.8%)	0(0%)	
**Repolarization**				
Minor	0(0%)	2(12.5%)	0(0%)	<0.01
Major	3(30%)	4(25%)	0(0%)	
**Arrhythmia**				
Minor	7(70%)	15(93.8%)	25(100%)	0.01
Major	1(10%)	2(12.5%)	0(0%)	
**Fatty tissue**				
Minor	0(0%)	0(0%)	0(0%)	<0.01
Major	4(40%)	3(18.8%)	0(0%)	
**RV image**				
Minor	0(0%)	4(25%)	0(0%)	<0.001
Major	4(40%)	6(37.5%)	0(0%)	
**Family history**				
Minor	2(20%)	2(12.5%)	0(0%)	0.01
Major	1(10%)	2(12.5%)	0(0%)	

ARVC: arrhythmogenic right ventricular cardiomyopathy, RV: right ventricle, RVOT: right ventricular outflow tract, VT: ventricular tachycardia.

Baseline demographic characteristics, electrocardiography, cardiac image study, electrophysiological study, and details of three-dimensional electroanatomic mapping and ablation results were reviewed retrospectively. All had 12-lead resting ECGs, 24-hour Holter examination, and two-dimensional (2D) echocardiography to obtain general laboratory data. Magnetic resonance imaging (MRI) was performed in 54 patients (76%), right ventriculography in 48 (68%), endomyocardial biopsy in 23 (32%), and coronary angiography in 56 (79%) of the enrolled subjects.

### Electrophysiological study, mapping, and radiofrequency catheter ablation

Antiarrhythmic drugs were discontinued for a minimum of five half-lives before catheter ablation (except amiodarone). In the absence of spontaneous VA, rapid ventricular pacing and programmed stimulation with up to three extra stimuli were performed with the catheter placed at the right ventricular apex and RVOT sequentially. If VA was still not inducible, intravenous isoprenaline 1–5 μg/min was infused to achieve at least 20% heart rate increment. If spontaneous VAs were not inducible during pharmacological provocation, the induction protocol was repeated. The QRS morphologies of spontaneous and/or induced VAs were compared with those of the documented VAs.

The localization of arrhythmogenic foci or critical isthmus sites were performed conventionally or by using a three-dimensional (3D) mapping system (CARTO 3.2 UDM version, Biosense Webster, Diamond Bar, CA, USA). For idiopathic VAs, activation mapping, defining the earliest local electrograms, and/or pacemapping aiming for at least 11 of 12 leads ECG matching with the clinical VAs were performed. For hemodynamically stable substrate VTs, activation mapping and entrainment mapping were performed for localization of the critical isthmus within a scar; pace mapping and/or substrate-based modification strategy, targeting the late and fractionated electrograms within a scar/low voltage zone during sinus rhythm or ventricular pacing, were used for unstable ventricular tachyarrhythmias. Repeat mapping was performed if suppression and/or elimination of idiopathic VA were not observed. An epicardial approach was performed according to ECG and electroanatomic mapping criteria after failed endocardial ablation [21,22].

RF energy was delivered at a temperature-controlled mode at 50–60°C with pulse duration of 60 seconds; maximal power was 50 W for a non-irrigated catheter and 30–35 Watts for an irrigated catheter, targeting for an impedance drop of 10 Ohms. If the idiopathic VA was suppressed within 30 seconds, RF energy would be maintained for a total of 60 seconds, and additional energy would be applied up to a maximum of 5 burns. For substrate VA ablation, we aimed at terminating the hemodynamic stable VT or elimination of all recorded abnormal electrograms from detailed mapping.

### Substrate analysis

Electrograms were filtered at 30 to 250 Hz and 1 to 240 Hz for bipolar and unipolar signals respectively. Three-dimensional RV/LV anatomies were created by using CARTO 3 system (MEM version, UDM module, Biosense Webster, Haifa, Israel) with a 3.5 mm open-irrigated tip catheter (Thermocool, Biosense Webster, CA, USA). During sinus rhythm mapping, a cut-off value of BV between 0.5 and 1.5 mV has been traditionally set up to define the endocardial substrates. Low voltage zone (LVZ) was defined by peak-to-peak bipolar voltage of 0.5 mV based on bipolar intracardiac recordings).[23] A cutoff value of unipolar voltage <5.5 mV was applied to define the unipolar LVZ.[22] In order to achieve homogeneously detailed maps, the fill threshold was set to 10 mm in areas with normal voltages and to 5 mm in areas with low-voltage amplitude. The voltage maps were edited by manually eliminating intracavitary points. The area of low voltage zone detection was calculated by the built-in commercialized software of the Carto system [24].

### ECG decomposition by principal component analysis (PCA)

Consider a data matrix X_*8xn*_, where each row corresponds to a standard ECG lead (I, II, V1, V2, V3, V4, V5, V6) and *n* is the number of the samples on each lead (*n*>>8). Note that only eight of the standard 12-lead ECG leads were used because of algebraic interdependency. The ECG matrix X_*8xn*_ was decomposed by the singular value decomposition of PCA to construct the orthogonal components in a signal space (File for details).[23] By PCA, the selected standard 8 ECG leads were reconstructed into an orthogonal 8-lead system. The first principal component accounts for most of the energy of QRS complex, while irregularity of QRS complex could be indicated by the contribution of the second and third principal components. By the fact that 99% of the ECG energy is included in the first three principal components,[23] only the vectors associated with the first three principal components were incorporated to investigate and describe the spatiotemporal variations of the QRS complex. Specifically, the first 3 principal components formed a 3-dimensional depolarization loop. Note that the remaining components relate to the components beyond the movement of QRS complex.

### QRS loop descriptor

**Fig 1:**
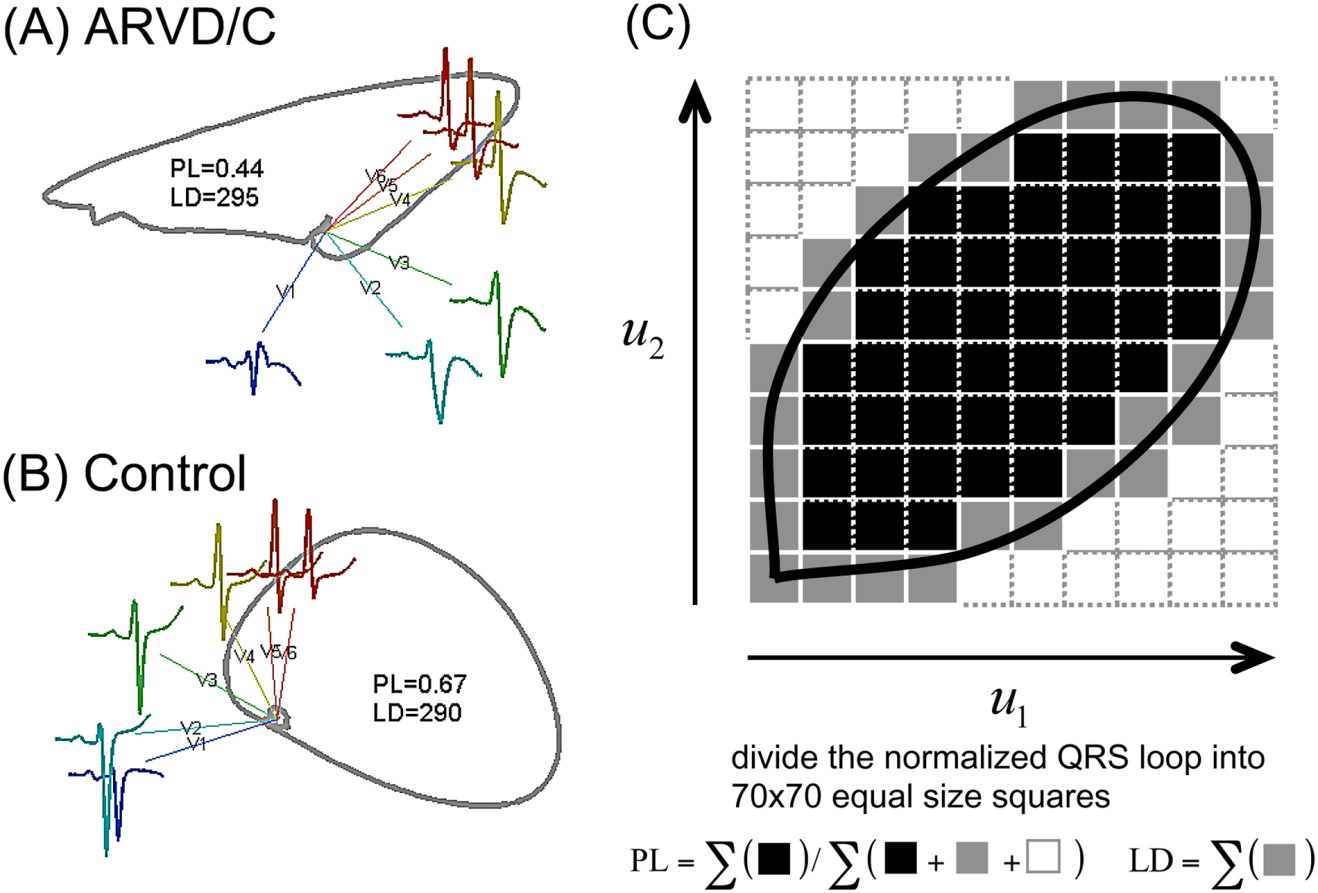
QRS loop of (A) ARVC and (B) control case and (C) loop descriptors. The QRS loop was constructed from the reconstruction vectors obtained after a principal component analysis. (ARVC: arrhythmogenic right ventricular cardiomyopathy; LD: loop dispersion; PL: loop area)

The 3-dimensional depolarization loop was mapped to a 2D space to characterize the depolarization regularity as shown in Fig 1, where the QRS loop in the 2D plane was constructed by taking the samples regarding the QRS complex of the first principal component as the x-coordinate and the corresponding samples of the second principal component as the y-coordinate, i.e. the planar QRS loop was spanned by vectors associated with the first two principal components.[23,25] A minimum rectangle that encompassed the planar loop was divided into N (N = 4900 in this study) small cells with equal size. The percentage of the cells inside the QRS loop, called the percentage of loop area (PL), indicated the shape and regularity of the QRS loop (Fig 1).[25] PL reduces with self-crossing areas and with convex and concave components in the loop. The total number of cells that the QRS loop passed indicated the loop dispersion (LD; Fig 1), which measures the variation of the QRS vector (i.e. the variation of the interlead relationship).[23] A larger LD suggested more homogeneity. Notice that the LD was abnormally large when the loop presented self-crossing areas.

The inter-lead QRS dispersion (IQRSD) indicated the dissimilarity of the shape between the QRS complexes of two ECG leads, based on the differences between two reconstructed vectors of orthogonal leads created from the 3-dimensional QRS loop. The difference was characterized by the angle between a pair of adjacent reconstruction vectors.[23,25] A small angle indicated spatially close vectors and similar QRS complex. The details were in the S1 File.

### Simulated ECG regarding depolarization abnormalities

Simulated ECG data were created by using a research tool, ECGSIM, where the electrical potentials on the body surface were derived by the transmembrane potentials on the myocardium according to the transfer function computed by the boundary element model [26]. The ECGSIM included the heart geometry (defined by 257 nodes) and torso model measured by MRI, and took into account the complex geometry and differences in the conductivity between the myocardium, blood pool, and lungs. A normal male’s heart geometry was used in the simulation. Ischemia was simulated by altering the shape of transmembrane potential, where the abnormal action potential was derived from the normal action potential by delaying the depolarization upstroke by 60 msec and reducing the action potential amplitude to two-thirds of the original level [27–29]. The adaptation of ischemia was linearly tapered off from 100% of the selected site to zero at the boundary of the designated area. The ischemic region was assumed to be approximately circular and its size was adjusted by the radius.

### Clinical follow-up

After discharge from the hospital, all patients were seen within 2 weeks, and further follow-up was scheduled every 3 months if no adverse events occurred. During the visit, the efficacy of the ICD therapy, and any adverse events, were assessed and analyzed. The use of antiarrhythmic drugs was determined by the clinical physicians.

### Statistical analysis

Data were expressed as numbers of count (percentage). The continuous variables were expressed as the mean ± standard deviation (SD). Baseline characteristics between the three groups (Group 1, Definite ARVC with epicardial ablation; Group 2, Definite ARVC without epicardial ablation; and Group 3, Idiopathic RVOT VT) were compared using one-way analysis of variance for continuous variables. The chi-square test with Yates' correction was used to analyze the categorical variables. A *P* value of <0.05 was considered statistically significant. Relationships between the proportion of LVZ and IQRSD were analyzed with a regression analysis, where the LVZ was an independent variable and the IQRSD was a dependent variable. All statistical analyses were performed using SPSS software version 17.0 (SPSS, IBM, Chicago, IL, USA).

## Results

### Patient characteristics

A total of 71 patients (mean age: 44±13 [20–68] years old, 25 [35.2%] men) were enrolled, including 51 patients with VT (Group 1–3) and 20 control patients (Group 4). Overall 26 (36.6%) patients had definite ARVC based on the revised TF criteria, 25 (35.2%) had RVOT VT without meeting the diagnosis of ARVC, and 20 (28.2%) had AVNRT (Group 4). From the demographic characteristics of these ARVC patients shown in Table 2, the baseline characteristics and MRI findings were comparable between the groups, and Group 1 had lower right and left ventricular ejection fractions. Substrate mapping showed more extension of the epicardial low voltage zone, and a longer total ventricular activation duration throughout the epicardium and endocardium.

**Table 2:** Baseline patients data.

Characteristics	Definite ARVC with epicardial ablation, Group 1 (N = 10)	Definite ARVC without epicardial ablation, Group 2 (N = 16)	Idiopathic RVOT VT, Group 3 (N = 25)	P value
**Underlying**				
Sex (male), N (%)	3(30%)	6(40%)	13(52%)	0.56
Age, N	50±15	44±15	46±15	0.78
ICD implants (%)	2(20%)	2(20%)	3(12%)	0.66
Inducible VT (%)	4(40%)	5(31.3%)	8(32%)	0.90
TF major criteria	1.1±0.93	1.2±1.05	0.66±0.97	0.39
TF minor criteria	1.5±0.92	1.2±0.43	1.5±0.87	0.54
**ECG factors**				
Epsilon wave	3(30%)	2(13%)	0(0%)	0.06
SAECG (+)	8(80%)	10(63%)	1(4%)	0.14
Image findings*				
RVEF (%)	38±13	46±13	49±13	0.05
LVEF (%)	40±20	50±11	60±7	0.04
**Cardiac MR findings**	10(100%)	16(100%)	13(52%)	0.52
Global RV dysfunction	2(20%)	3(18.8%)	0(0%)	0.10
Regional RV dysfunction	4(40%)	5(31.3%)	2(8%)	0.31
RV regional wall thinning bulging/aneurysm	1(10%)	4(25%)	2(8%)	0.60
Fat deposition by MRI	4(40%)	4(25%)	1(4%)	0.37
**Substrate mapping** ¶	10(100%)	16(100%)	25(100%)	0.99
Total activation time (Epi-Endo)	240±145	148±48	139±49	0.03
Unipolar LVZ (endo, cm2)	56±28.8	41.5±19.3	30.1±18. 9	0.14
Unipolar LVZ (endo%)	23±11.3	17.9±9.79	15.9±10.3	0.39
Bipolar LVZ (endo, cm^2^)	23.4±19.9	16.1±12.82	12.4±8.35	0.26
Bipolar LVZ (endo%)	10.1±7.73	7.23±6.33	6.64±4.54	0.43
Unipolar LVZ (epi, cm^2^)	177±45.2	43.5±18.7	0±0	<0.001
Unipolar LVZ (epi%)	56.7±12	18.7±5.41	0±0	<0.001

EF: ejection fraction, ICD: implantable cardioverter defibrillator, LV: left ventricle, RV: right ventricle, RVOT: right ventricular outflow tract; TF: Task Force, VF: ventricular fibrillation, Epi: epicardium, Endo: endocardium;

*Assessed by echography, MRI, or angiography during intervention.

^¶^ Two group 2 patients and one group 3 patient received electroanatomic epicardial mapping

### Clinical outcome

During a mean follow-up of 24±23 months, no patients died from cardiovascular diseases/or fatal arrhythmias. One patient (1.41%) underwent heart transplantation due to ARVC progression. For the ARVC patients requiring epicardial ablation in Group 1, all 10 patients (100%) experienced malignant events, including 2 (20%) with a fast VT recorded by the ICD (cycle length of <250 milliseconds) and 10 (100%) with hemodynamically unstable ventricular arrhythmias (VAs), and all patients (10/10, 100%) received defibrillator therapy as primary or secondary treatment. In Group 2, 3 patients (18.8%) experienced a short cycle length VT (less than 250 milliseconds) and manifested with syncope. In the univariate analysis, a definite ARVC diagnosis, lower RV ejection fraction, lower left ventricular ejection fraction, pathological change, ECG repolarization abnormality, and the presence of an epicardial unipolar LVZ, were related to the rapid VT events in all patients. In the multivariate analysis, the presence of an epicardial unipolar LVZ and definite ARVC diagnosis were independent predictors (odds ratio [OR]: 11.3, 95% confidence interval [CI]: 1.55–84.5, *P* = 0.02, and OR: 18.9, 95% CI: 0.9–407.9, *P* = 0.05, respectively).

### ECG analysis and QRS descriptors

**Fig 2:**
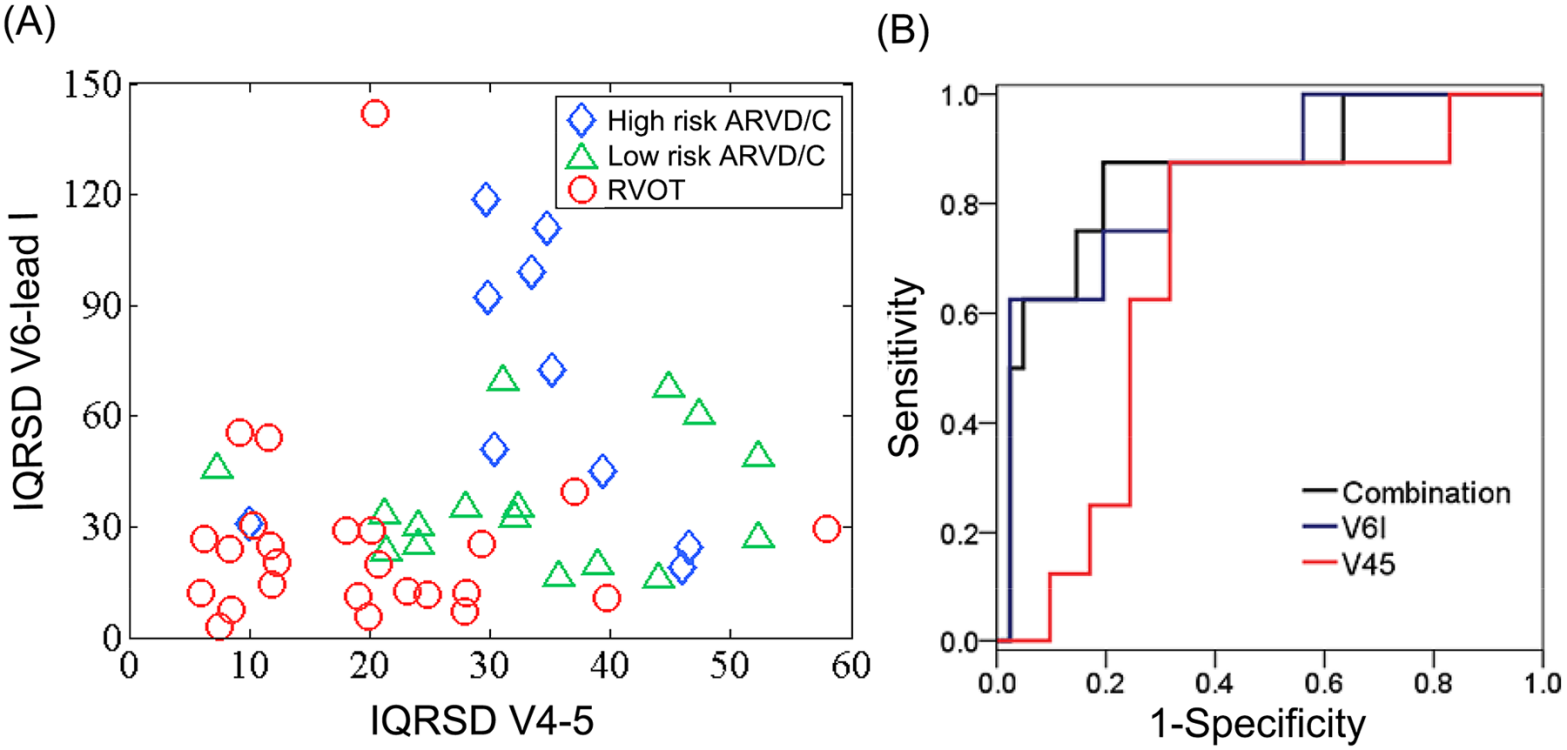
(A) Scatter plot and (B) ROC curves of the IQRSD between V4-V5 and between V6-I. The IQRSD between V4-V5 separated definite ARVC from RVOT VT (borderline cases), while the IQRSD between V6-1 differentiated Group 1 Group 2. Right panel: ROC curves of IQRSD between V4-V5 and between V6-I. The area under the curve further improved after a combination of the IQRSD between V4-V5 and V6-I. (IQRSD: interlead QRS dispersion; ROC: receiver-operator characteristic).

The 12-lead ECG analysis showed that the QRS duration, R wave amplitude, and S wave amplitude were similar between the ARVC (fulfilling the TF criteria) and RVOT-VT patients (not fulfilling the TF criteria, p>0.05, not shown). Fig 1 showed the QRS loop and analysis in patients with ARVC and control patients and the loop descriptors. Regarding the QRS loop descriptors, only the PL in the ARVC group was significantly smaller than in the control patients (Table 3, p<0.05). On the other hand, the LD exhibited no difference among the groups (Table 3). Regarding the QRS descriptors, the IQRSD between V1 and V2 was capable of differentiating VAs from the RV (Group 1 to Group 3) from the control (*P*<0.01). Definite ARVC (Group 1 and 2) exhibited a greater IQRSD between V4 and V5 than the idiopathic RVOT-VT or controls (Group 3 or 4, *P* = 0.001). In addition, the group 1 had a greater IQRSD between V6 and lead I than the control (*P* = 0.001). Notably, definite ARVC (Group 1 and 2) led to a consistently larger IQRSD between the V1-V2, V4-V5, and V6-I pairs as compared to Group 3. A receiver operatoring characteristic (ROC) analysis suggested that the combination of the IQRSD between V4-V5 and V6-lead I attained the best sensitivity (87.5%) and specificity (80.5%) to detect ARVC requiring epicardial ablation (Group 1) from those of Group 2 (Fig 2).

**Table 3:** Analysis of QRS parameters in different types of patients.

Inter-lead QRS Dispersion	Definite ARVC with epicardial ablation (N = 10, Group 1)	Definite ARVC without epicardial ablation (N = 16, Group 2)	Idiopathic RVOT-VT patients (N = 25, Group 3)	Control patients (AVNRT, N = 20, Group 4)	P value
Lead V1-V2	50±25*	70±21*	50±29*	38±22	<0.01
Lead V2-V3	56±14	47±19	54±18	54±11	0.78
Lead V3-V4	41±12	49±14	52±14	42±11	0.66
Lead V4-V5	34±10**	33±12**	19±12	18±11	0.001
Lead V5-V6	31±19	23±11	21±10	20±13	0.39
Lead V6- I	66±37**	36±21	26±27	24±22	0.001
PL	0.59±13*	0.56±0.14*	0.64±0.10	0.67±0.05	0.02
LD	298±24	309±42	291±12	293±9	0.15

Deviation between leads was calculated according to the 3D projection of the electrogram vectography (range 0–180 degree);

* P<0.05, Post-Hoc analysis, compared with Group 4;

** P<0.05, Post-Hoc analysis, compared with Group 3, Group 4.

### Cardiac MR findings and ECG analysis

In our patients, cardiac MR was performed in all patients with ARVC (10/10 and 16/16, 100%, for Group 1 and 2), however, only 52% and 0% were performed in patients with Group 3 and 4. The QRS loop descriptor PL was smaller in all patients with focal dyskinesia, as compared to patients without dyskinesia (0.456±0.210 vs. 0.622±0.107, P = .026); PL was larger in patients with generalized RV dyskinesia. Also, the IQRSD between V1-V2 was higher in patients with RV free wall focal bulging 79±19 vs 52±29 degree (P = 0.03). Furthermore, LD was higher in patients with generalized RV dysfunction (333.3±77.4 vs. 294.76±19.1, P = .022). The other factors were similar in patients with or without structural abnormality identified by cardiac MR.

### Detection of an epicardial unipolar low-voltage zone with interlead morphology dispersion

**Fig 3:**
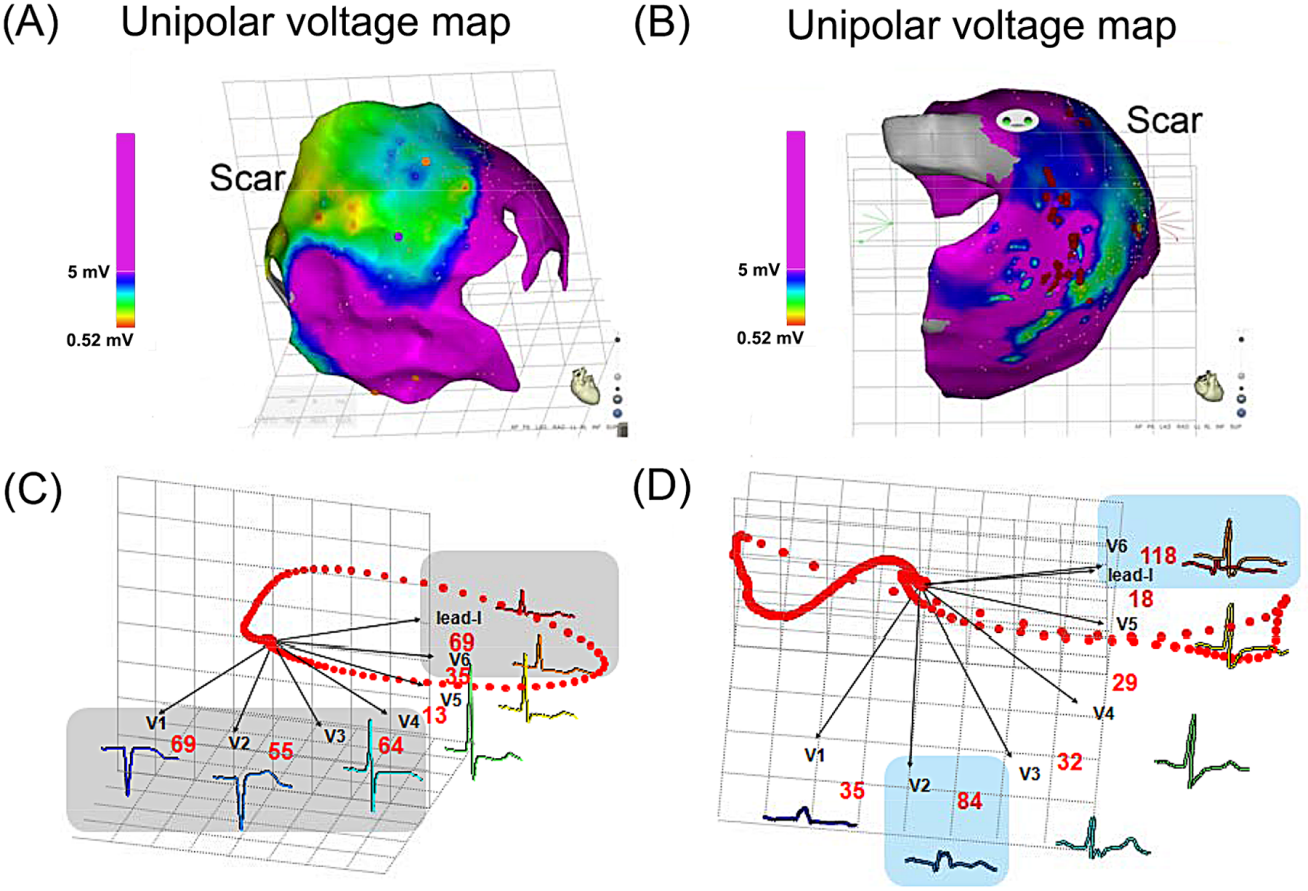
(A, B) 3D electroanatomic map and (C, D) corresponding reconstruction vectors of precordial ECG (lower panel) in two ARVC patients. The spatial inhomogeneity between leads V1 and V2 (C) and between leads V6 and I (D) indicate corresponding epicardial unipolar scar (green area, less than 5.5 mV) at the right ventricle site and left lateral left ventricle, respectively.

**Fig 4:**
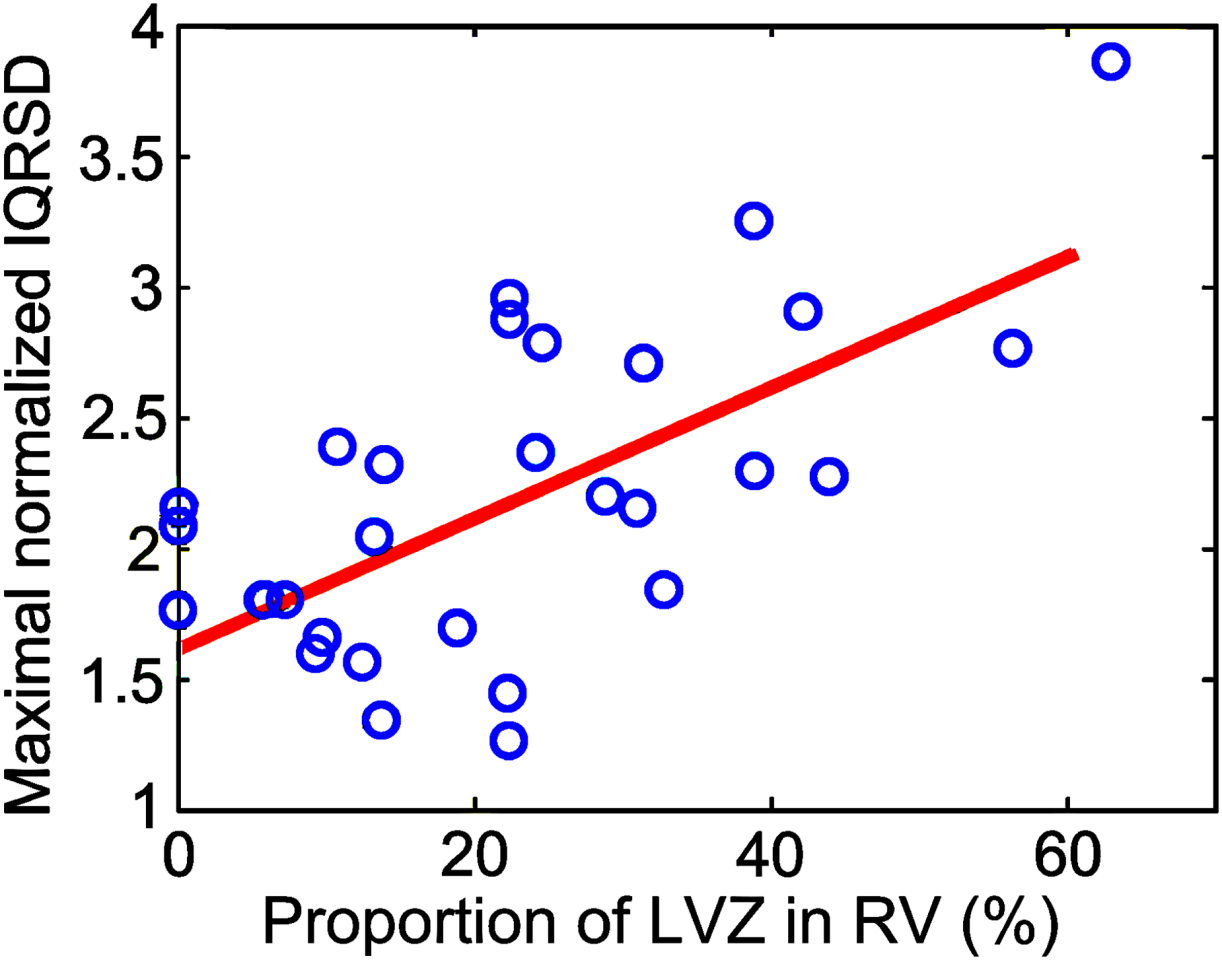
Relationship between IQRSD and LVZ in clinical data. Positive correlation between maximal normalized IQRSD and proportion of the LVZ in the RV. (LVZ: low-voltage zone; RV: right ventricle)

As shown in the left panel of Fig 3, the epicardial unipolar mapping revealed a diffuse LVZ in the right anterior free wall, which was associated with dispersion among precordial leads V1-V3. Moreover, an epicardial unipolar LVZ involving an extensive part of the right and left free wall region was associated with a lateral extension of the lead V6 and lead I region of the precordial leads, as demonstrated in the right panel of Fig 3. Furthermore, by analyzing the ECG in patients during 3D electroanatomic mapping, the maximal normalized IQRSD among the precordial lead pairs was also correlated with the proportion of unipolar LVZs in the RV (γ = 0.62, *P*<0.001). Note that the IQRSD in these patients was normalized to the median of the IQRSD pairs in each patient to eliminate any individual difference (Fig 4).

### Simulated ECG regarding a local substrate abnormality in the computer model

**Fig 5:**
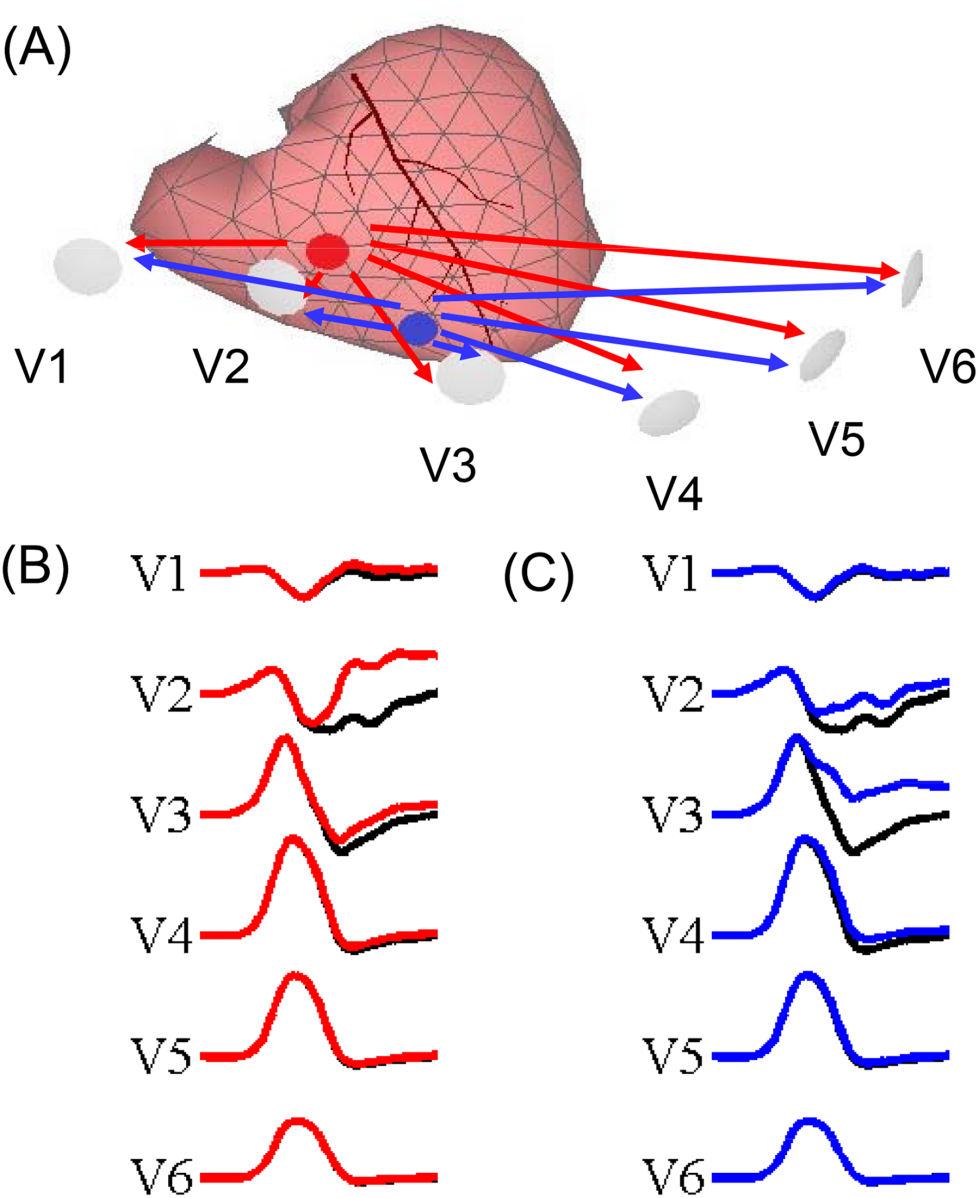
Illustration of the change in the QRS morphology and associated IQRSD in computer simulation with respect to ischemia under lead V2 (red dot) and under and between V3 and V4 (blue dot). (A) heart geometry; (B) QRS changes in precordial leads with ischemia under lead V2; and (C) QRS changes in precordial leads with ischemia under and between V3 and V4. The black lines in (B) and (C) indicate the normal precordial ECG. We delayed the depolarization upstroke of the action potential by 60 msec and reduced the amplitude to two-thirds of the original level to simulate ischemic myocardium as the red and blue traces. When the ischemia was under V2, the IQRSD of V1-V2 and V2-V3 dramatically increased from 48.3 to 66.4 degrees and from 45.1 to 87.6 degrees, respectively. When the ischemia was under and between V2 and V3, the IQRSD of V2-V3 and V3-V4 dramatically increased from 45.1 to 68.4 degrees and from 58.7 to 81.8 degrees, respectively.

**Fig 6:**
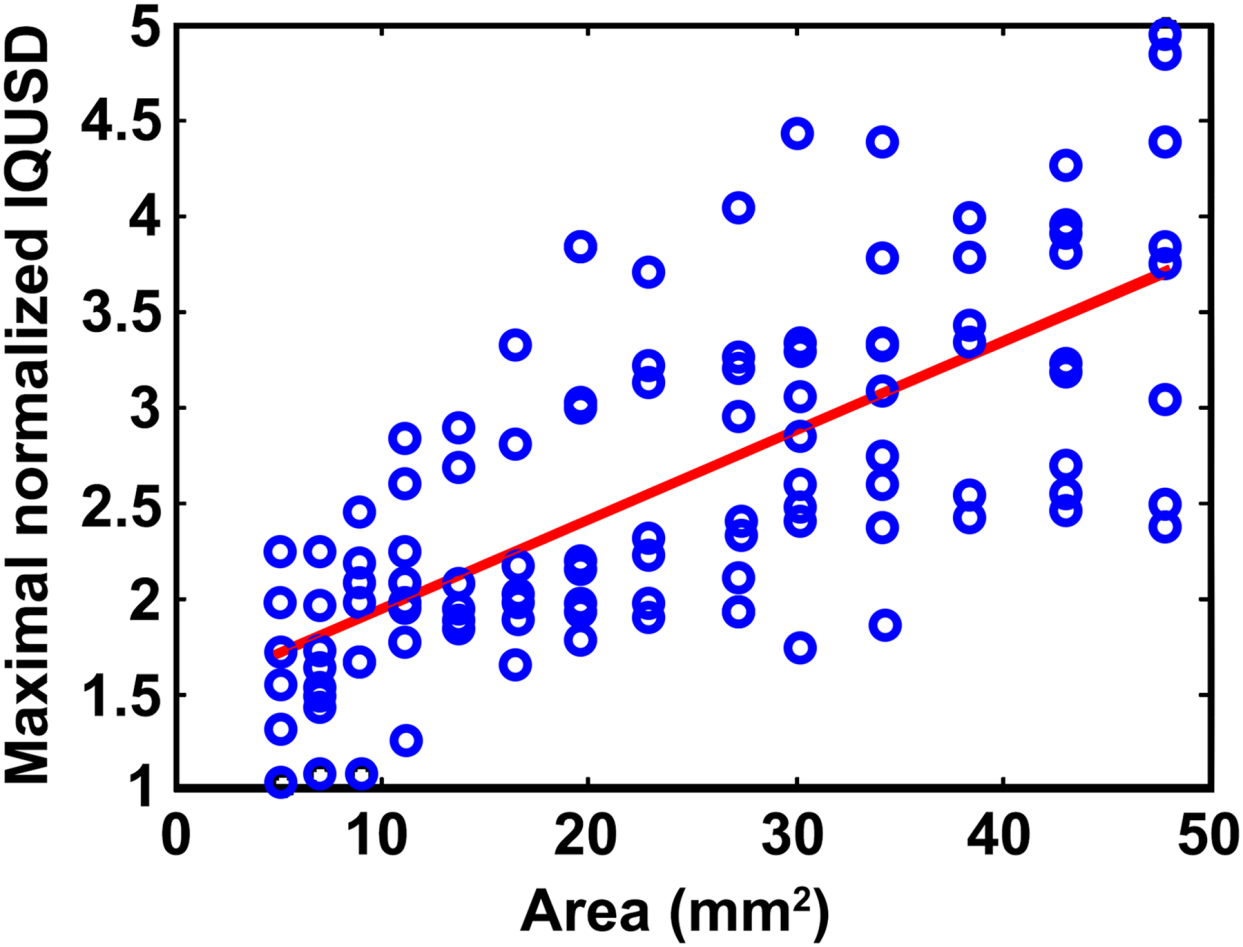
Computation simulation for IQRSD with respect to the proportion of abnormal substrate. Positive correlation between maximal normalized IQRSD and the area of abnormal substrate in the epicardium.

In the computer simulation model, an ischemic area of 2.5 cm^2^ was assigned to the projected epicardial region below lead V2. The QRS morphology of V2 changed substantially as illustrated in Fig 5, which subsequently led to an increase in the IQRSD of the V1-V2 pair. When the abnormal substrate area was moved to the projected epicardial region under and between leads V2 and V3, the QRS morphology of V2 was slightly restored, but the V3 changed substantially and the IQRSD of the V2-V3 and V3-V4 pairs increased. We further used the simulated ECG from 9 sites that were uniformly distributed in the middle of the ventricles to investigate the effect of a local abnormal substrate. All pairs with a greater IQRSD in the simulated precordial ECG leads were associated with a corresponding abnormal substrate (anatomically nearest region) with respect to the corresponding pair with a normal IQRSD. The maximal normalized IQRSD among adjacent precordial lead pairs was positively correlated with the size of the ischemic area (γ = 0.71, *P*<0.001; Fig 6).

## Discussions

### Main findings

With the use of PCA, the IQRSD derived from reconstructed vectors of the 12-lead ECG effectively diagnosed and differentiated ARVC from idiopathic RVOT-VT, which suggested its ability to reflect the regional conduction abnormalities in the ventricle. The prediction of the PCA relevant precordial leads was also confirmed by a computer simulation model: All pairs with IQRSD in the simulated precordial ECG were associated with a corresponding abnormal substrate beneath the precordial leads. In addition, we found that the maximal normalized IQRSD among adjacent precordial lead pairs was positively correlated with the size of the abnormal substrate. The degree of dispersion among the surface ECG leads could be applied to estimate the proportion of the epicardial abnormal substrate prior to the ablation procedure, as a noninvasive survey.

### Diagnostic criteria of ARVC

The diagnosis of ARVC is mostly based on classical or modified Task Force criteria, because either ECG depolarization abnormality or positive MRI finding may lead to low sensitivity or specificity to diagnose the patients with ARVC (Table 2). This is compatible with previous study [30]. The ECG of ARVC patients usually shows a wider QRS duration (>110 milliseconds) in lead V1 and an inversion of T waves in precordial leads V1-V3 during sinus rhythm. The extent of the T wave inversion in the precordial leads beyond lead V1 correlates with the extent of RV involvement [31]. Prolongation of the QRS duration in the right precordial leads reflects late conduction in the RV [32]. Small electrical potentials occurring at the end or immediately after the QRS complex in leads V1 or V2, which are called Epsilon waves, were observed in approximately 30% of ARVC patients who had VT. Since the publication of these criteria, a number of ECG markers of ARVC have been proposed to detect the delayed RV activation in the right precordial leads, which include the presence of parietal block, slurred S waves, and an increased QRS duration of precordial leads [33–35]. In addition to morphological abnormalities of ECG, a comprehensive clinical evaluation of ARVC based on other TF criteria is also warranted to assess the arrhythmic risk [36]. In this study, our data further showed that ARVC altered the regularity of the QRS loop, as indicated by the decreased PL compared to the control patients (Fig 1).

### Comparison of RVOT-VT and ARVC

RVOT-VT comprises a subgroup of idiopathic VT that occurs in the absence of structural heart disease and epicardial scar on echocardiography, angiography, or MRI. In the 12-lead ECG, the mean QRS duration was longer in ARVC patients compared to the RVOT-VT group in all the leads. Recent studies identified certain features of the QRS complex during VAs that can facilitate distinguishing ARVC from idiopathic RVOT-VT [37–39]. Hypothetically, these conduction abnormalities would result in a greater delay from earliest onset to local onset of the QRS complex, greater duration of the QRS complex, and irregularities of conduction manifesting as notching of the QRS complex.

Our data demonstrated that the regularity of the QRS loop was different between the two groups and the IQRSD between V1 and V2 differentiated the patients with VAs from control patients, which suggested that the depolarization abnormalities associated with VAs from RV were reflected by the large dispersion between the two leads. In addition, The IQRSD between V4 and V5 showed the capability to diagnose ARVC in patients with RVOT-VT. When ARVC progresses, the substantial conduction block might occur in ventricles, greatly altering the depolarization regularity. As a result, IQRSD between V6 and I became abnormally large even though both leads have similar lateral views of the ventricles.

Previous study also demonstrated the high inter-observer variability in the assessment of epsilon wave[40]. Therefore, the present results also provided a subjective and quantitative measurement of abnormal electrical activation in patients with ARVC. Additionally, previous studies showed that electrical abnormalities can frequently be found preceding the detectable structural changes in ARVC, reflecting the pivotal role of ECG assessment as the initial clinical manifestation [41]. Furthermore, previous study also proved that cardiac magnetic resonance is less sensitive and might not be sufficient for the identification of RV scar [42]. The above evidence proved the results and concepts of current results by recognizing the abnormal ECG features in ARVC.

### IQRSD and LVZ

A previous study indicated that the dispersion of ventricular depolarization in ARVC patients indicated a VT recurrence and risk of sudden cardiac death [18,19]. Our study further demonstrated that the IQRSD of the precordial leads indicated the progression of ARVC. Substrate mapping also demonstrated an extensive epicardial LVZ and the requirement for epicardial ablation. Both the clinical and simulated data exhibited a similar correlation between the maximal normalized IQRSD and the proportion of LVZ, which suggested the capability of predicting the extent of the LVZ for ablation by using the IQRSD (Figs 4 and 6); and the maximal IQRSD may indicate the location of the abnormal substrate in the RV (Figs 3 and 5).

### Clinical implication

The IQRSD derived from reconstructed vectors of the surface ECG effectively identified the potential abnormal substrate beneath the precordial leads of surface ECG. QRS features of the surface ECG can be used to distinguish ARVC from other idiopathic ventricular arrhythmias or control patients, which supports its effectiveness over the conventional ECG determination criteria, such as a QRS duration or the T wave inversion. It is also worth of mentioning that the QRS feature analyses could be useful noninvasive techniques to identify the scar for patients with implantable cardiac defibrillators while commonly used MRI scanning is not practical for patients with defibrillators.

## Limitation

The study has several limitations. First, not all diagnostic tests were performed in every enrolled proband. Second, reconstruction of the IQRSD derived from the PCA may not completely be compatible with the 3D location of the precordial leads. However, the computer simulation model demonstrated that the IQRSD indicated the location of the corresponding epicardial scar, which confirmed the clinical observation. Third, in this study, we only include patients with prior subjects were evaluated by ECG, Holter monitoring, signal-averaged ECG, invasive electrophysiological study/ablation, and cardiac MRI. Therefore, the number of patients is limited. Fourth, the application of dispersion of IQRS to distinguish ARVC from RVOT-VT required further validation. Furthermore, early stage ARVC not-fulfill the Task Forth criteria might present as RVOT-VT. Further prospective trials measuring the dispersion of IQRS before catheter ablation and long-term follow-up trials with IQRS dispersion measurement for the disease progression in suspicious early stage ARVC patients are warranted.

## Conclusions

With the use of PCA, the IQRSD derived from reconstructed vectors effectively diagnosed ARVC with potential abnormal substrate noninvasively. Increased dispersion between a pair of precordial leads indicated the probability of epicardial abnormal substrate in the region beneath those precordial leads, and the possibility of epicardial ablation in ARVC patients. The extent of abnormal substrate in the diseased myocardium could be estimated by the spatial heterogeneity in the depolarization of surface ECG as noninvasive procedure.

## Supporting information

S1 File Supplementary material of methodology. (DOCX)
