# Supplementary material for: A novel noninvasive surface ECG analysis using interlead QRS dispersion in arrhythmogenic right ventricular cardiomyopathy
Source: PLoS One. 2017 Aug 3;12(8):e0182364. doi: 10.1371/journal.pone.0182364 (PMC5542590; doi:10.1371/journal.pone.0182364)
Supplement: S1 File — (DOCX) [file pone.0182364.s001.docx]

**Supplementary material**

**Methods**

*ECG decomposition by principal component analysis (PCA)*

Consider a data matrix , where each row corresponds to a standard ECG lead (I, II, V1, V2, V3, V4, V5, V6) and *n* is the number of the samples on each lead (*n*>>8). Note that only eight of the standard 12-lead ECG leads were used because of algebraic interdependency. The ECG matrix was decomposed by PCA to extract the orthogonal components in a signal space. To be more specific [1],

(1)

where, the value of $\text{λ}_{\text{i}}$ was associated with the ECG energy along with the corresponding vectors in the left unitary matrix $\mathbf{U}$.

Mathematically, each ECG lead could be reconstructed by

. (2)

where $\vec{\mathbf{v}}_{\text{i}}=\{\text{v}_{\text{i,1}},\text{v}_{\text{2,i}},\ldots,\text{v}_{\text{n,i}}\}$.

The principal components of the data matrix $\mathbf{X}_{8xn}$ projecting on the space spanned by $\vec{\mathbf{u}}_{\mathbf{i}}$ were given by

${P_{m,n}=\left[ \begin{matrix} \vec{p_{1}} \\ \vdots\\ \vec{p_{m}} \end{matrix} \right]=SV^{T}=\left[ \begin{matrix} \begin{matrix} \lambda_{1} & 0 & 0 \\ 0 & \lambda_{2} & 0 \\ 0 & 0 & \lambda_{3} \end{matrix} & & \\ & \ddots& \\ & & \lambda_{m} \end{matrix} \right]\left[ \begin{matrix} V_{11} & \cdots& V_{1n} \\ \vdots& \ddots& \vdots\\ V_{n,1} & \cdots& V_{\mathrm{nn}} \end{matrix} \right]}^{T}$ (3)

where $\vec{\mathbf{s}}_{i}$’s are the principal components of $\mathbf{X}_{8xn}$ and are orthogonal to each other.

By the fact that 99% of the ECG energy is included in the first three principal components [1], only the vectors associated with the first three principal components were incorporated to investigate and describe the spatiotemporal variations of the QRS complex.

*Interlead QRS dispersion*

The inter-lead QRS dispersion (IQRSD) indicated the dissimilarity of the shape between the QRS complexes of two ECG leads, which was characterized by the angle between the reconstructed vectors associated with the first three principal components [1,2]. The reconstructed vectors were denoted by

, (4)

where was used to equalize the energy in the direction of each vector. Electrophysiological abnormalities of the QRS complex may be subtle regarding the depolarization dispersion caused by regional scar or ischemia (see the illustration in the simulated ECG in the Results.). Equalization manipulation of the energy of each reconstructed vector would further obscure the abnormalities within the QRS complex because the energy of the reconstructed vector pointing in the major depolarization direction was several orders larger than the vectors associated with the depolarization dispersion. In other words, a reconstructed vector pointing in the major depolarization direction had a dominant influence on the QRS dispersion. Generally, an alteration in the major depolarization path required a substantial portion of conduction abnormalities in the ventricular substrate and was not the case in the early stage of ARVC. To detect regional depolarization abnormalities reliably, we quantified the IQRSD between adjacent pairs of precordial leads, and the energy of the reconstructed vectors of the QRS was unscaled by normalization of the corresponding energy factor in the matrix , i.e.

, (5)

Where the resultant reconstructed vectors were relevant to the original unitary matrix $\mathbf{U}$ with truncation of the first three components.

Then IQRSD was defined by the angle between two reconstructed vectors of a pair of adjacent ECG leads as given by

$\hat{\theta_{i,j}}=\cos^{-1}\left( \frac{{(u}_{i1}u_{j1}+u_{i2}u_{j2}+u_{i3}u_{j3})}{\sqrt{{(u_{i1})}^{2}+{(u_{i2})}^{2}+{(u_{i3})}^{2}}\sqrt{{(u_{j1})}^{2}+{(u_{j2})}^{2}+{(u_{j3})}^{2}}} \right)$ . (6)

A small value of $\hat{\text{θ}}_{\text{i,j}}$ indicated that the reconstructed vectors were close to each other and that the QRS morphology on the associated i and j leads were similar.

*Simulated ECG regarding depolarization abnormalities*

Simulated ECG data was created by using a research tool, ECGSIM, where the electrical potentials on the body surface were derived by the transmembrane potentials on the myocardium according to the transfer function computed by the boundary element model [3]. The ECGSIM included the heart geometry (defined by 257 nodes) and torso model measured by MRI, and took into account the complex geometry and differences in the conductivity between the myocardium, blood pool, and lungs. A normal male’s heart geometry was used in the simulation. Ischemia was simulated by altering the shape of transmembrane potential, where the abnormal action potential was derived from the normal action potential by delaying the depolarization upstroke by 60 msec and reducing the action potential amplitude to two-thirds of the original level [4-6]. The adaptation of ischemia was linearly tapered off from 100% at the selected site toward zero at the boundary of the designated area. The ischemic region was assumed to be approximately circular and its size was adjusted by the radius.

*Clinical Follow-up*

After discharge from the hospital, all patients were seen within 2 weeks, and further follow-up was scheduled every 3 months if no adverse events occurred. During the visit, efficacy of the ICD therapy, and any adverse events, were assessed and analyzed. The use of antiarrhythmic drugs was determined by the clinical physicians.

**References**

1. Acar B, Yi G, Hnatkova K, Malik M (1999) Spatial, temporal and wavefront direction characteristics of 12-lead T-wave morphology. Med Biol Eng Comput 37: 574-584.

2. Zabel M, Acar B, Klingenheben T, Franz MR, Hohnloser SH, et al. (2000) Analysis of 12-lead T-wave morphology for risk stratification after myocardial infarction. Circulation 102: 1252-1257.

3. van Oosterom A, Oostendorp TF (2004) ECGSIM: an interactive tool for studying the genesis of QRST waveforms. Heart 90: 165-168.

4. Kleber AG, Janse MJ, van Capelle FJ, Durrer D (1978) Mechanism and time course of S-T and T-Q segment changes during acute regional myocardial ischemia in the pig heart determined by extracellular and intracellular recordings. Circ Res 42: 603-613.

5. Miller WT, 3rd, Geselowitz DB (1978) Simulation studies of the electrocardiogram. II. Ischemia and infarction. Circ Res 43: 315-323.

6. Galeotti L, Strauss DG, Ubachs JF, Pahlm O, Heiberg E (2009) Development of an automated method for display of ischemic myocardium from simulated electrocardiograms. J Electrocardiol 42: 204-212.
